# Establishment of a proteome profile and identification of molecular markers for mouse spermatogonial stem cells

**DOI:** 10.1111/jcmm.12407

**Published:** 2014-10-29

**Authors:** Quan Zhou, Yueshuai Guo, Bo Zheng, Binbin Shao, Min Jiang, Gaigai Wang, Tao Zhou, Lei Wang, Zuomin Zhou, Xuejiang Guo, Xiaoyan Huang

**Affiliations:** State Key Laboratory of Reproductive Medicine, Department of Histology and Embryology, Nanjing Medical UniversityNanjing, China

**Keywords:** spermatogonial stem cells, self-renew, proteome, nucleoproteins, surface marker

## Abstract

Spermatogonial stem cells (SSCs) are undifferentiated cells that are required to maintain spermatogenesis throughout the reproductive life of mammals. Although SSC transplantation and culture provide a powerful tool to identify the mechanisms regulating SSC function, the precise signalling mechanisms governing SSC self-renewal and specific surface markers for purifying SSCs remain to be clearly determined. In the present study, we established a steady SSC culture according to the method described by Shinohara's lab. Fertile progeny was produced after transplantation of cultured SSCs into infertile mouse testis, and the red fluorescence exhibited by the culture cell membranes was stably and continuously transmitted to the offspring. Next, *via* advanced mass spectrometry and an optimized proteomics platform, we constructed the proteome profile, with 682 proteins expressed in SSCs. Furthermore bioinformatics analysis showed that the list contained several known molecules that are regulated in SSCs. Several nucleoproteins and membrane proteins were chosen for further exploration using immunofluorescence and RT-PCR. The results showed that SALL1, EZH2, and RCOR2 are possibly involved in the self-renewal mechanism of SSCs. Furthermore, the results of tissue-specific expression analysis showed that *Gpat2* and *Pld6* were uniquely and highly expressed in mouse testes and cultured SSCs. The cellular localization of PLD6 was further explored and the results showed it was primarily expressed in the spermatogonial membrane of mouse testes and cultured SSCs. The proteins identified in this study form the basis for further exploring the molecular mechanism of self-renewal in SSCs and for identifying specific surface markers of SSCs.

## Introduction

Spermatogenesis is an intricate and coordinated process by which thousands of spermatozoa are produced per second throughout the 24-hr day within the testis. Like other adult self-renewing tissues that rely on stem cells for the replenishment of differentiated cells at a constant rate or more rapidly after toxic injury, continual spermatogenesis is dependent on an adult tissue-specific stem cell population called spermatogonial stem cells (SSCs). SSCs arise from gonocytes postnatally and have the unique ability to undergo self-renewal division and to support spermatogenesis throughout the life span of an animal 1,2. Similar to other tissue-specific stem cells, SSCs are rare and comprise only 0.03% of all germ cells in rodent testes 2,3. Considerable efforts have been directed towards the development of techniques for assaying SSC activity (SSC transplantation) and for maintaining SSCs *in vitro* (SSC culture), and when combined, SSC transplantation and culture can provide a powerful tool to identify the mechanisms regulating SSC function.

In 1994, the germ cell transplantation technique was developed and provided the first functional assay for SSCs 4. With regard to SSC culture, in 2003, the Japanese research team comprising Kanatsu-shinohara *et al*. succeeded in the long-term culture of SSCs from neonatal mouse testis, and named these cells germline stem (GS) cells. These cell lines continued to proliferate for at least 2 years and restored fertility to congenitally infertile recipient mice following transplantation into seminiferous tubules 5. This group then modified and improved the culture system and developed serum- and feeder-free culture conditions for GS cells 6. Another prominent group leading stem cell culture research is the team at Brinster's lab from the University of Pennsylvania: they enriched SSCs *via* antibody selection with the specific surface protein THY-1 and cultured SSCs from pup mouse testis that could restore fertility when transplanted into infertile recipients 7. Similar cultures were subsequently established from SSCs of adult mice by other groups 8,9. Because of the establishment of stable culture systems, it is now possible to study SSCs in detail.

Self-renewal is an important feature of SSCs in *in vitro* culture. In recent years, researchers have been of the opinion that this process is regulated by both extrinsic environmental stimuli and specific intrinsic gene expression. For example, glial cell line-derived neurotrophic factor (*gdnf*) is expressed in Sertoli cells in the testis and is reported to be a critical extrinsic growth factor that stimulates self-renewal in rodent SSCs 10–12. With regard to intrinsic molecular pathways regulating SSC self-renewal, specific transcription factors, such as *Bcl6b*, *Etv5*, *Lhx1*, *MilI*, *Nanos2* and *Plzf*, have received much attention 13–18. However, the precise signalling mechanisms governing SSC self-renewal remain to be investigated.

SSCs form a small fraction of spermatogonia and are morphologically indistinguishable from committed progenitors, so the discovery of SSC surface markers will not only improve SSC identification but also contribute to an understanding of the mechanism of self-renewal division. The first SSC surface markers identified were integrin α6 and β1 19. Furthermore, *Thy-1* (*CD90*), *CD9*, *Epcam* and *Gfra1* have been identified as molecules expressed on SSCs 20–23. However, although several SSC surface markers have been identified using the spermatogonial transplantation technique, none of these molecules are specifically expressed on SSCs and novel specific surface markers need to be identified.

In the present study, based on the method reported by Shinohara's lab 5, we established a stable SSC cell line and transferred it to infertile males who were able to produce fertile progeny. Moreover, we established and compared the proteome profiles of MEF (mouse embryonic fibroblast) feeder cells and mouse SSCs co-cultured with MEFs; we identified 682 proteins specifically expressed in SSCs. Further bioinformatics analysis showed that among these proteins were several molecules that are known to be regulated in SSCs. Based on this, several nucleoproteins and membrane proteins were chosen for further investigation, to identify the highly expressed proteins, and thereby elucidate the molecular mechanism of self-renewal and determine more specific surface markers of SSCs.

## Materials and methods

### Animals

All animal experimentation protocols were approved by the ethics committee of Nanjing Medical University (China). Testis cells were collected from a 2-day neonatal transgenic mouse line (B6.129(Cg)-*Gt(ROSA)26Sor*^*tm4(ACTB-tdTomato,-EGFP)Luo*^/J; The Jackson Lab, Bar Harbor, ME, USA) that was bred into the ICR background (Lab Animal Center of Nanjing Medical University, Nanjing, China). These mice express cell membrane-localized red fluorescence in widespread cells/tissues, so donor SSCs can be readily identified following transplantation. The testis cells were digested with 1 mg/ml collagenase (type IV; Sigma-Aldrich, St. Louis, MO, USA) for 15 min., and this was followed by digestion with 0.25% trypsin EDTA (1×) (Invitrogen, Carlsbad, CA, USA) with 1.4 mg/ml DNase (Sigma-Aldrich) for 10 min.

After culture (culture conditions described below), the cultured cells were transplanted into B6.129 mice inbred with ICR, which is the same strain as the donor mice but without fluorescence in any cells. To eliminate endogenous spermatogenesis, these mice were treated with busulfan (50 mg/kg) at 6 weeks of age 4 and transplantation was performed 1 month later.

### Culture conditions

The culture conditions were those described by Shinohara *et al*. 5. Briefly, dissociated testis cells were cultured on a 0.2% (w/v) gelatin-coated tissue culture plate (2 × 10^5^ cells/3.8 cm^2^) overnight. The plates were washed twice with PBS before use. The floating cells were passaged to secondary plates. These cells were then passaged two or three times before they were transferred to medium containing MEFs. The culture medium for the testis cells was StemPro-34 SFM supplemented with StemPro supplement (Invitrogen), 25 μg/ml insulin, 100 μg/ml transferrin, 60 μM putrescine, 30 nM sodium selenite, 6 mg/ml d-(+)-glucose, 1 μl/ml dl-lactic acid, 5 mg/ml bovine albumin (Sigma-Aldrich), 1 mM minimal essential medium (MEM) sodium pyruvate solution, 2 mM GlutaMaxTM supplement, 0.05 mM 2-mercaptoethanol, MEM vitamin solution, MEM nonessential amino acid solution (Invitrogen), 10^−4^ M ascorbic acid, 10 μg/ml d-biotin, 30 ng/ml beta-estradiol, 60 ng/ml progesterone (Sigma-Aldrich), 20 ng/ml mouse epidermal growth factor (EGF) (Millipore Corporation, Billerica, MA, USA), 10 ng/ml bovine fibroblast growth factor basic (bFGF), 15 ng/ml recombinant rat glial cell line-derived neurotrophic factor (GDNF) (R&D Systems, Minneapolis, MN, USA) and 1% foetal bovine serum (Invitrogen). The cells were maintained at 37**°**C in an atmosphere containing 5% carbon dioxide.

### Immunohistochemical staining for SSC markers

The three primary antibodies used against SSC markers were based on previous reports: goat anti-OCT3/4 (sc8629; Santa Cruz Biotechnology, Santa Cruz, CA, USA), mouse anti-PLZF (OP128L; Millipore Corporation) and goat anti-GFRA1 (GT15004; Neuromics, Edina, MN, USA). Mouse anti-SSEA1 (sc-101462; Santa Cruz Biotechnology) was used as a negative marker because SSEA-1 is an early germline development pluripotent marker primarily expressed in primordial germ cells 24. For the controls, the primary antibodies were replaced with normal goat or mouse IgG. After they were washed with PBS, the cells were incubated with FITC-labelled secondary antibody (Beijing ZhongShan Biotechnology, Beijing, China). The nucleus was stained with 5 μg/ml Hoechst H33342 (Sigma-Aldrich), and the samples were analysed under a ZEISS LSM 710 (Carl Zeiss, Oberkochen, Germany).

### Transplantation and analysis of fluorescence in the progeny

To briefly remove the MEF feeders from co-culture, the cell suspension was plated on empty plates for approximately 3 hrs. The MEF cells rapidly attached to the plate, after which the floating cells were collected for transplantation. Approximately 6–8 μL of the donor cell suspensions at a concentration of 1 × 10^8^ cells/mL was injected into the seminiferous tubules of busulfan-treated recipient testis 4,5. Two months after transplantation, some of the recipient mice were killed for analysis, and one recipient mouse was mated with two adult female ICR mice. The resultant F1, F2 and F3 male offspring were mated with two adult female ICR mice, respectively. The F1, F2, F3 and F4 progeny were observed under a DFP-1 dual fluorescent protein flashlight (NightSea, Bedford, MA, USA).

### Sample preparation for mass spectrometry

MEF feeder cells and mouse SSCs co-cultured with MEF (to enrich SSCs, the cell suspension was plated on empty plates for approximately 3 hrs) were dissolved in 7 M urea, 2 M thiourea, 65 mM DTT, and 1% (v/v) protease inhibitor cocktail, and then centrifuged at 40,000×g for 1 hr at 4°C, respectively. Protein concentrations were measured by the Bradford assay, and 85 μg of each sample was reduced, alkylated and sequentially digested with modified trypsin (Promega Corporation, Madison, WI, USA). These in-solution digests were loaded onto a strong-cation exchange column (1 mm ID, 10 cm long, packed with Poros 10 S; DIONEX, Sunnyvale, CA, USA) for fractionation. A linear salt gradient in the form of ammonium formate in 5% ACN was applied at a flow rate of 50 μL/min. Mobile phase A comprised 95:5 H2O:ACN and 5 mM ammonium formate buffer (pH + 2.7), and mobile phase B comprised mobile phase A and 800 mM ammonium formate (pH + 2.7). The gradient used was 0–56% B for 20 min., 56–100% B for 1 min., 100% B for 5 min., 100% to 0% B for 1 min. and 0% B for 20 min. before the next run. In each series of experiments, 100-μL fractions were collected every 2 min., and 20 fractions were obtained in total.

### Mass spectrometric analysis and database search

For capillary reverse-phase LC and mass spectrometric analysis, each fraction was directly loaded onto a μ-precolumn™ cartridge (0.3 × 5 mm, 5 μm, 100 Å; DIONEX) at a flow rate of 20 μl/min. The trap column effluent was then transferred to a reverse-phase microcapillary column (0.075 × 150 mm, Acclaim® PepMap100 C18 column, 3 μm, 100 Å; DIONEX). The reverse-phase separation of peptides was performed with buffer A (2% ACN and 0.5% acetic acid) and buffer B (80% ACN and 0.5% acetic acid); a 122-min ACN gradient was used (4% to 7% buffer B for 3 min., 7% to 33% buffer B for 102 min., 33% to 50% buffer B for 10 min., 50% to 100% buffer B for 3 min., 100% buffer B for 3 min., 100% to 4% buffer B for 1 min.). Peptide analysis was performed with LTQ Orbitrap Velos (ThermoFisher Scientific, San Jose, CA, USA) coupled directly to an LC column. An MS survey scan was obtained for the m/z range 350–1800, and MS/MS spectra were acquired from the survey scan for the 20 most intense ions (as determined by Xcalibur mass spectrometer software in real time). Dynamic mass exclusion windows of 60 sec. were used, and siloxane (m/z 445.120025) was used as an internal standard.

RAW files for LC-MS/MS identifications were processed using MaxQuant (version: 1.3.0.5, Max Planck Institute of Biochemistry, Martinsried, Germany), and identified with the Andromeda search engine. The peak lists were searched against the UniProtKB mouse proteome sequence database (Updated: May, 2012), which contains 55,269 entries. Carbamidomethylation of cysteine (+57 D) was set as a fixed modification, and oxidization of methionine (+16 D) was set as a variable modification. The initial mass tolerances for protein identification from MS and MS/MS peaks were 20 p.p.m. and 0.5 D, respectively. Two missed cleavages were permitted, and full cleavage by trypsin was used. The false discovery rates (FDR) of the identified peptides and proteins were estimated by searching against the database with the reverse amino acid sequence. Only peptides with a minimum of six amino acids and an FDR of 1% were considered for identification.

### SSC proteome annotation

We identified proteins expressed in MEFs and proteins expressed in SSCs co-cultured with MEFs. After removing the common proteins expressed in the two cell types, we constructed the SSC proteome profile. The Uniprot entries were converted to Entrez Gene IDs. The Entrez Gene IDs were loaded onto the Web-based Gene Set Analysis Toolkit (http://bioinfo.vanderbilt.edu/webgestalt/) to identify the hyper-represented WikiPathways.

### Bioinformatics analysis

To further explore the significance of the identified proteins, the Pathway Studio (v6.0) software (Ariadne Genomics, Rockville, MD, USA), a specialized graph visualization engine, was used to determine the relevant molecular functions of the proteins. The gene list was imported into Pathway Studio to identify the cell process, which was confirmed *via* the PubMed/Medline hyperlink embedded in each node. Subsequently, all proteins were loaded onto the Database for Annotation, Visualization and Integrated Discovery (DAVID) 25 to identify their subcellular localization. To explore known and predicted protein–protein interactions, all the nucleoproteins were uploaded into Search Tool for the Retrieval of Interacting Genes/Proteins (STRING) (v9.05) 26. The BioGPS database 27 (a free extensible and customizable gene annotation portal that is a useful resource for learning about gene and protein function) was used to determine the gene expression level of membrane proteins in different mouse tissues.

### Verification of nucleoproteins and membrane proteins *via* cell immunofluorescence

From the nucleoprotein and membrane protein lists, we validated the expression of several proteins involved in stem cell function. SSCs were cultured on MEF feeders in a Millicell EZ slide (Millipore Corporation). SALL1, EZH2, RCOR2 and PLD6 were chosen for further exploration based on previous reports. The following commercial antibodies were used: rabbit anti-SALL1 (ab31526; Abcam, Cambridge, MA, USA), mouse anti-EZH2 (612666; BD Bioscience, San Jose, CA, USA), mouse anti-RCOR2 (612146; BD Bioscience) and rabbit anti-PLD6 (ab170183; Abcam). For the controls, the primary antibodies were replaced with normal rabbit or mouse IgG. After being washed with PBS, the samples were incubated with FITC-labelled secondary antibody (Beijing ZhongShan Biotechnology). The nucleus was stained with 5 μg/ml Hoechst H33342 (Sigma-Aldrich). All samples were observed under a ZEISS LSM 710 (Carl Zeiss).

### Analysis of tissue distribution of membrane proteins *via* RT-PCR and whole-mount staining with seminiferous tubules

We analysed the tissue expression patterns of membrane proteins identified *via* the BioGPS database. Several membrane genes highly or uniquely expressed in the mouse testis were chosen for validation *via* RT-PCR. cDNA from 11 mouse tissues (testis, heart, brain, thymus, stomach, spleen, liver, lung, kidney, ovary and uterus) was PCR-amplified with specific primers (Table1), and mouse *Gapdh* was used as the control.

**Table 1:** Sequences of specific primers for the ten membrane proteins amplified

Gene name	Sequence
*Gpat2*	F: 5′-CAGCCCATTGTGCTAGGTGA-3′
	R: 5′-AGGACCACACCCTTTTGGTG-3′
*Pld6*	F: 5′-GCGCAAGGGATACAGGTACG-3′
	R: 5′-GACGTTCTCCCGGTTGTTCT-3′
*Letm2*	F: 5′-CAAGCCCTCAGCCTACCAAA-3′
	R: 5′-GTTCTCAACAGCCTTCGCCT-3′
*Fam118a*	F: 5′-AAGACGAAGGTTCTTCAGTGGG-3′
	R: 5′-AGTACAAGAAGAGCGCCTGG-3′
*Reep6*	F: 5′-CCTTTCTACTACGCGGGCAA-3′
	R: 5′-TACTTGGCGTCCCGGGTTAT-3′
*Dnajc18*	F: 5′-ACAGCAAAAGTCGGAGCTGA-3′
	R: 5′-AAGGACTGGAGGGGACAAGT-3′
*Bbs9*	F: 5′-CTTCCGAGGGATCCATGTCG-3′
	R: 5′-ACTGCTGTGGTCTCTTGGTC-3′
*Rhbdd3*	F: 5′-AAAGCTGAATGCGACCCAGA-3′
	R: 5′-AGCCGTAACACTCCTTGCAG-3′
*Dync2h1*	F: 5′-CCTCGTAGCCAGAACCCTTG-3′
	R: 5′-CTGTTGCCCTTGCAGTTTCC-3′
*Tmem39b*	F: 5′-GTTCTCGAAGCAGGACCACT-3′
	R: 5′-TGCACGAACAGGGCTATGAG-3′
*Gapdh*	F: 5′-AGTGGCAAAGTGGAGATTGTT-3′
	R: 5′-GTCTTCTGGGTGGCAGTGAT-3′

For whole-mount staining, seminiferous tubules of mouse testis tissues were dissected at post-partum day 2.5 and 28. The prepared seminiferous tubules were fixed with 4% paraformaldehyde (PFA) in PBS for 30 min., washed three times with PBS for 15 min. each time, and then blocked with 5% BSA for 2 hrs at room temperature. Following incubation with rabbit anti-PLD6 (ab170183; Abcam) and goat anti-PLZF (AF2944; R&D Systems) overnight at 4°C, tubules were incubated with AlexaFluor 488-labelled donkey anti-rabbit IgG and AlexaFluor 555-labelled donkey anti-goat IgG (Invitrogen) at a 1:1000 dilution for 2 hrs at room temperature. For negative controls, the primary antibody was replaced with normal rabbit and mouse IgG. The nucleus was stained with 5 μg/mL Hoechst H33342 (Sigma-Aldrich) for 30 sec. All samples were observed under a ZEISS LSM 710 microscope (Carl Zeiss).

## Results

### *In vitro* culture of mouse SSCs

**Fig 1:**
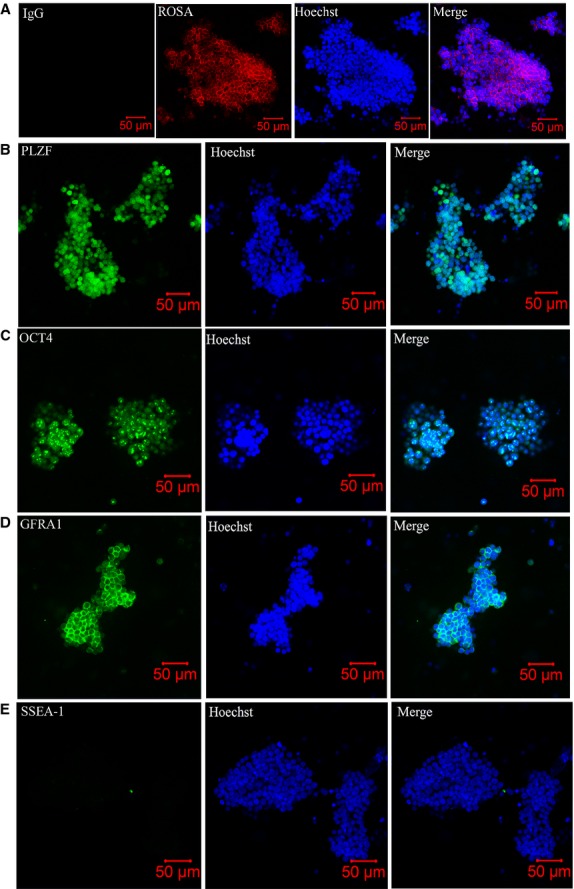
Morphology and phenotypic characterization of spermatogonial stem cell (SSC) colonies from neonatal mouse testis cells. (A) Cells at the 31st passage exhibited red fluorescence at their membrane. (B–D) Most SSCs expressed high levels of three known markers of undifferentiated cells: PLZF, OCT4 and GFRA1. (E) The negative control SSEA1 was not detected in the cultured SSCs. Hoechst indicates nuclear staining.

We established a stable *in vitro* culture of mouse SSCs according to the method described by Shinohara *et al*. 5. The mouse model used in this study was established using B6.129 mice inbred with ICR mice. By 4 weeks, the cultures were in a relatively steady-state and continued to generate colonies of compacted clusters of cells with unclear borders and exhibiting red fluorescence (Fig.1A). The cell line was passaged every 6–7 days for a total of 32 times and stored till analysis. One batch was frozen at the 11th passage for 2 months and is still in use for culture.

### Phenotype of SSCs

To evaluate the phenotype of the cultured cells, several undifferentiated SSC markers were characterized by indirect immunostaining at the 11th and 18th passage. As shown in Figure1B and D, most cells stained positively for PLZF, OCT4 and GFRA1. PLZF and OCT4 were expressed as nucleoproteins and GFRA1 was expressed as a membrane protein in cultured SSCs. The cells were completely negative for SSEA-1 (PGC marker) (Fig.1E) 24. These results indicate that the majority of cells had an undifferentiated spermatogonial cell phenotype.

### SSC transplantation

**Fig 2:**
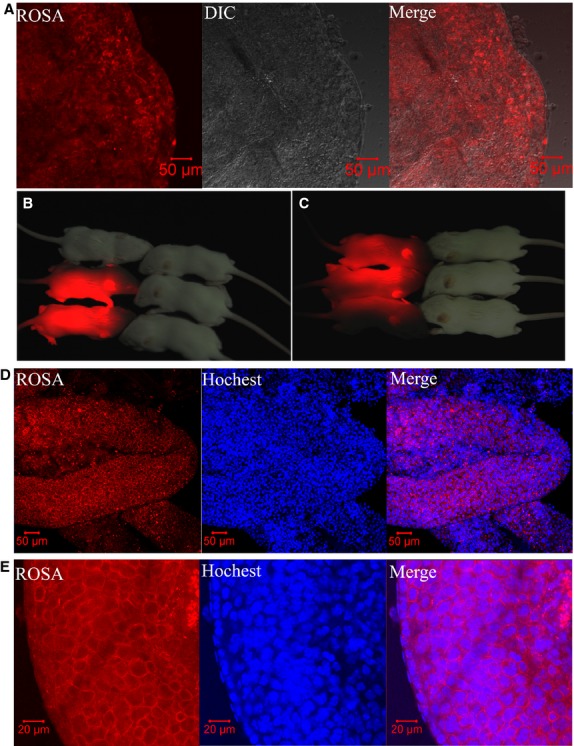
Spermatogenesis regeneration and offspring production after spermatogonial stem cell (SSC) transplantation. (A) The SSCs with membrane fluorescence were settled and incorporated with germ cells of the recipient testis in the injected seminiferous tubules. (B) Red fluorescence was observed in 27% of the pups. (C) Half of the progeny expressed red fluorescence. (D and E) Histological appearance of the recipient testis showing normal spermatogenesis with red membrane fluorescence of spermatogenic cells in some seminiferous tubules. Hoechst indicates nuclear staining.

To determine the stem cell activity of cultured cells, we performed spermatogonial cell transplantation. Approximately 5 × 10^5^ cells cultured for about 180 days (20th passage) were injected into each testis of two busulfan-treated recipient adult mice. Three days after transplantation, one of the recipient mice was killed and its testes were used for immunofluorescence analysis. In the injected seminiferous tubules, the SSCs were found to have settled and incorporated with germ cells of the recipient testis (Fig.2A). The other recipient mouse was mated with ICR females 2 months after transplantation to determine whether fertility was restored. Table2 shows detailed information about the mating procedure. The results showed that the red fluorescence was transmitted to 27% of the pups (Fig.2B). At the time of writing this manuscript, we had obtained the F1, F2 and F3 progeny, half of which expressed red fluorescence (Fig.2C).

**Table 2:** Detailed information showing the mating procedures of the receipt mice transplanted with cultured SSCs

	Number of female ICR mice	Number of offspring	Number of pups exhibiting red fluorescence	Red fluorescence pups/total pups (%)
First mating	3	19	6	31.58
Second mating	2	14	3	21.43
Third mating	2	14	4	28.57
Fourth mating	2	12	3	25
Total	9	59	16	27.12

Nine months after transplantation, we killed the recipient mouse for morphological analysis. The results showed that some seminiferous tubules were stained red in the testis. The red-stained tubules were excised for confocal analysis, and the germ cell membranes were also found to be red (Fig.2D and E).

### Peptide and protein identification

We constructed large-scale proteomes for MEF feeder cells and mouse SSCs co-cultured with MEFs by using 2D-LC followed by LTQ Orbitrap Velos identification. For reliable proteomic identification, we selected high-scoring peptide sequences with an FPR of 1% as determined by Maxquant 28. Redundant proteins or protein isoforms that could not be differentiated from each other on the basis of MS/MS data were presented as a unique protein group. As a result, more than 6700 proteins and 6500 proteins were identified in MEFs and SSCs co-cultured with MEFs, respectively. We focused on the proteins that were only expressed in SSCs and finally constructed the proteome profile for SSCs using 682 proteins (Table S1).

### Bioinformatics analysis of the identified SSC proteome

**Fig 3:**
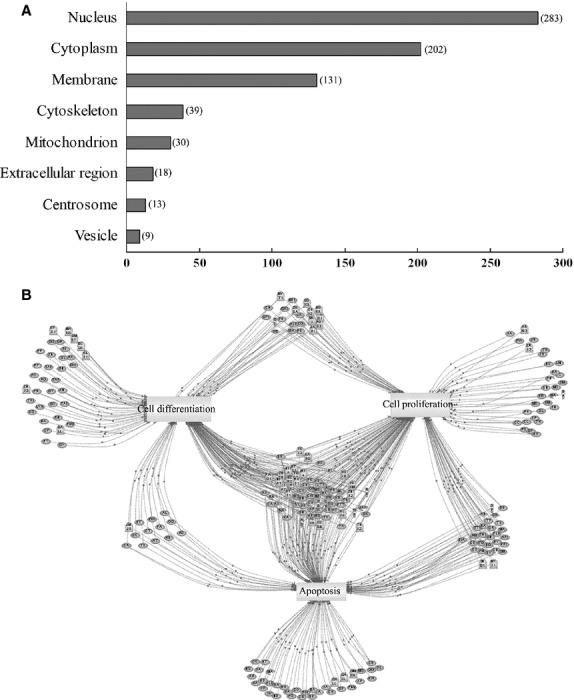
Subcellular distribution and functional annotation of specific proteins in mouse spermatogonial stem cells (SSCs). (A) Subcellular distribution of the 682 proteins in the SSC proteome was based on gene ontology annotations by DAVID. The largest proportion of proteins identified was in the nucleus (283), followed by the cytoplasm (202), membrane (131) and other subcellular structures. (B) Functional annotations by the Pathway Studio software. Cell processes are denoted by arrows: 161 proteins (Ellipse) were involved in cell proliferation events; 147 proteins, cell differentiation events and 159 proteins, apoptosis events.

The subcellular localization of each identified protein was based on a previous report 25. When an individual protein was known to be localized in more than one cellular compartment, all of the localizations were counted non-exclusively. Figure3A shows the cellular distribution of the identified proteins. The largest proportion of proteins was found in the nucleus (283), followed by the cytoplasm (202), cell membrane (131) and other subcellular structures.

Pathway annotation of the SSC proteome by Pathway Studio revealed that 161 proteins played a role in cell proliferation events; 147 proteins, cell differentiation events and 159 proteins, apoptosis events (Fig.3B). The results indicated that the cultured SSCs depicted active self-renewal and differentiation potential.

**Fig 4:**
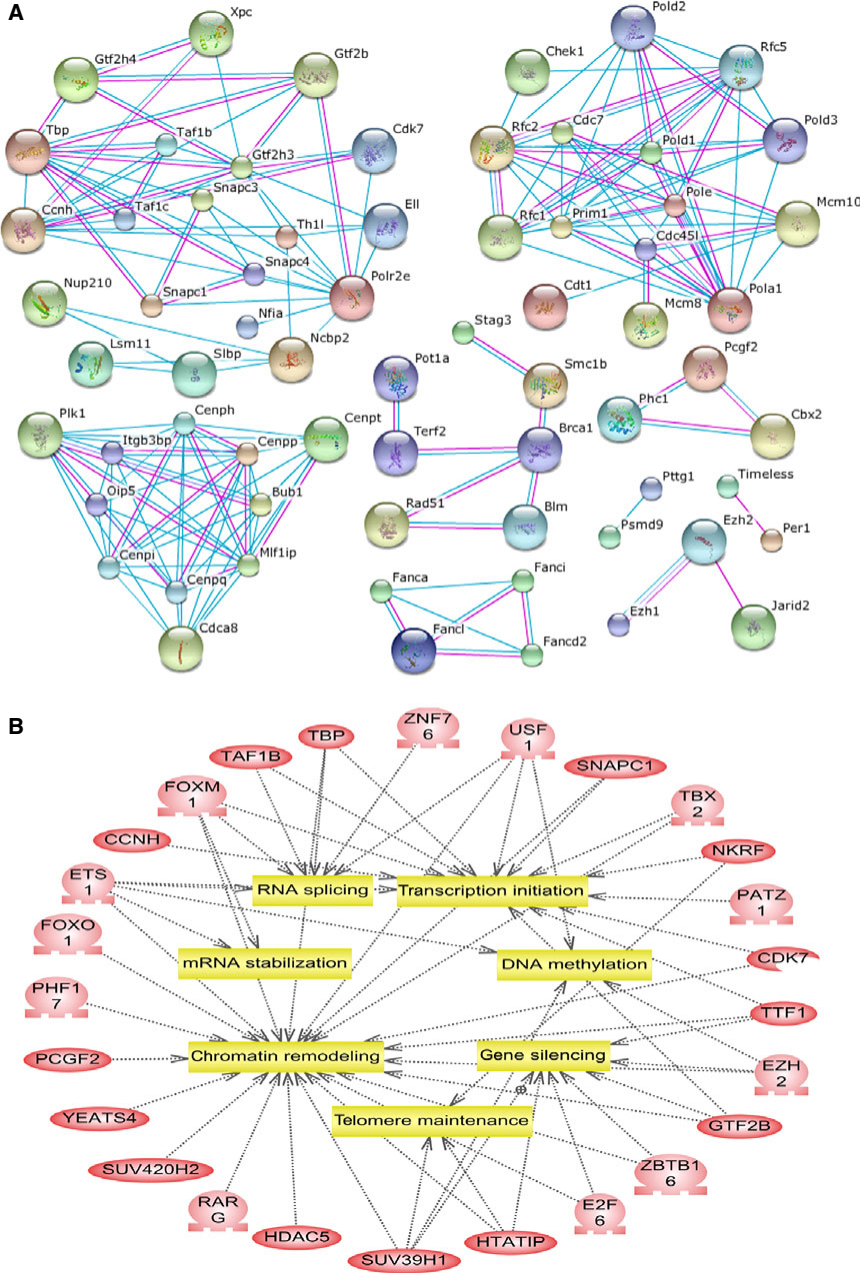
Interaction networks and important cellular events of the predicted nucleoproteins. (A) The interactions are presented as networks joining the nucleoproteins, using the STRING database. (B) The relations between nucleoproteins and cellular events involved in the nucleus were analysed *via*DAVID and 26 proteins were reported to participate in RNA splicing, transcription initiation, DNA methylation, gene silencing and so on.

To obtain detailed information about nucleoprotein interactions, the STRING database was used to show the known interactions derived from high-throughput experiments. In Figure4A, the interactions are depicted as networks connecting the nucleoproteins. Furthermore, the relations between nucleoproteins and cellular events involved in the nucleus are described in Figure4B (from DAVID): 26 proteins were found to participate in RNA splicing, transcription initiation, DNA methylation, gene silencing and so on.

### Comparison with known transcriptomes of mouse SSCs

**Fig 5:**
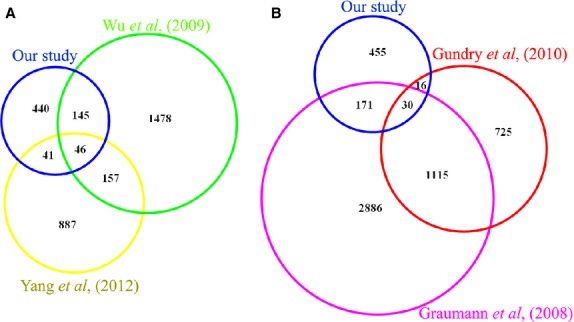
The published transcriptome of enriched mouse germ cells (A) and proteome of mouse embryonic stem cells (B) were compared with our proteome. The proteins were converted to Ensembl Genes, and overlapping genes (A) and proteins (B) are shown in the Venn diagrams.

When compared with previous reported transcriptomes of mouse SSCs, 191 genes matched the ones reported in the gene expression profiles of mouse gonocytes by Wu *et al*. 29; 87 matched the ones in the gene expression profile of mouse SSCs by Yang *et al*. 30 (Fig.5A); and 46 genes were present in all the three profiles.

### Verification of several nucleoproteins in mouse SSCs

**Fig 6:**
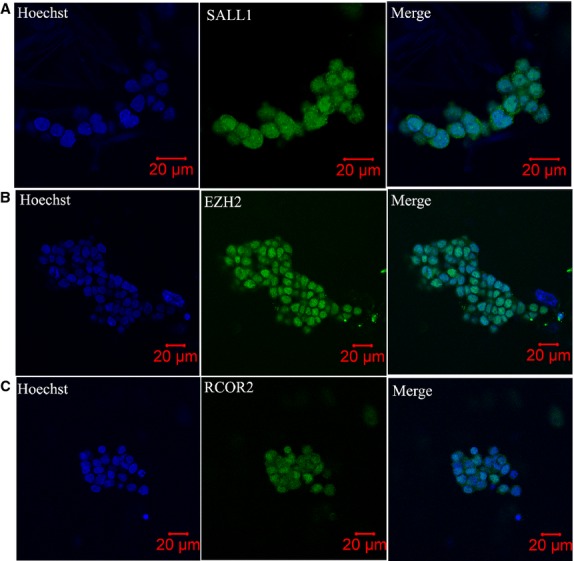
Immunolocalization of SALL1 (A), EZH2 (B) and RCOR2 (C) in cultured spermatogonial stem cells (SSCs). All three proteins were highly expressed in the nucleus of SSCs. Hoechst indicates nuclear staining.

We compared the proteome identified in our study with known published proteomes of mouse embryonic stem cells (ESCs) 31,32. As shown in Figure5B, 46 proteins were identified both in our study and the study of Gundry *et al*. 31; 201 proteins were identified both in our study and that of Graumann *et al*. 32; and 30 proteins were identified across all three studies. Among the 30 proteins, three with high-quality antibodies – SALL1, EZH2 and RCOR2 – were chosen for further verification. As shown in Figure6, all three proteins were highly expressed as nucleoproteins in cultured SSCs. The results indicated that SALL1, EZH2 and RCOR2 might be important for SSC self-renewal and maintenance of pluripotency.

### Tissue distribution of membrane proteins

**Fig 7:**
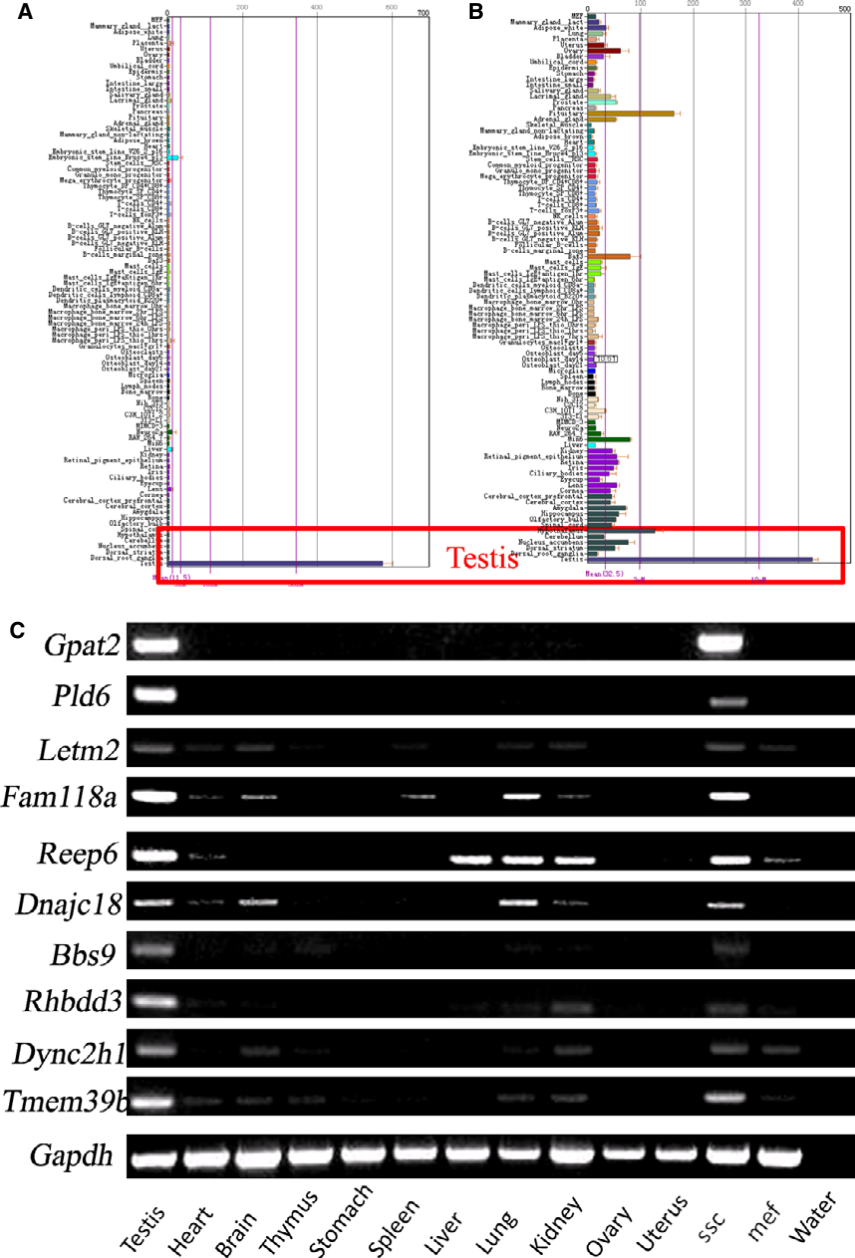
Tissue distribution of membrane proteins as determined by RT-PCR. (A and B) Tissue expression patterns of membrane proteins *via* the BioGPS database showed that the proteins were uniquely and highly expressed in mouse testis. (C) cDNA from 11 mouse tissues (testis, heart, brain, thymus, stomach, spleen, liver, lung, kidney, ovary and uterus) were PCR-amplified with specific primers. Among them, *Gpat*2 and *Pld6* were identified as being uniquely expressed in mouse testis and cultured spermatogonial stem cells (SSCs). *Letm2*, *Fam118a*, *Reep6*, *Dnajc18*, *Bbs9*, *Rhbdd3*, *Dync2h1* and *Tmem39b* were widely but highly expressed in mouse testis and SSCs.

The BioGPS database was used to predict the expression level of the 131 membrane proteins (analysed *via* DAVID) in multiple mouse tissues. Several proteins that were uniquely or highly expressed in mouse testes (Fig.7A and B) were chosen for further validation *via* RT-PCR. Among them, *Gpat2* and *Pld6* were identified as being uniquely expressed in mouse testes and cultured SSCs. *Letm2*, *Fam118a*, *Reep6*, *Dnajc18*, *Bbs9*, *Rhbdd3*, *Dync2h1* and *Tmem39b* were widely but highly expressed in mouse testes and SSCs (Fig.7C).

**Fig 8:**
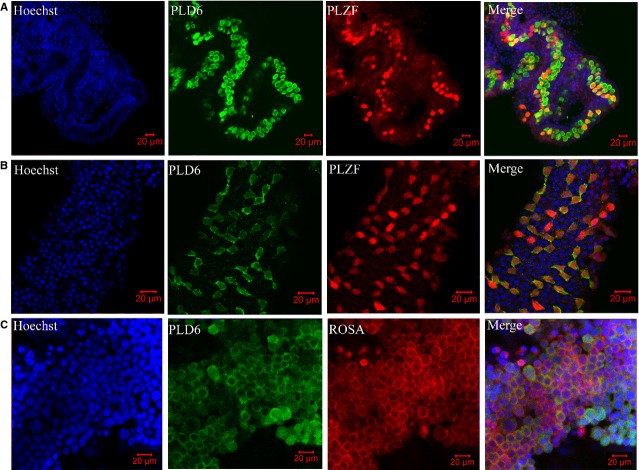
Cellular localization of PLD6 in post-partum day 2.5 (A), and day 28 (B) of mouse testis and (C) in cultured spermatogonial stem cells (SSCs) *via* immunofluoresence. PLD6 was uniquely expressed on the cell membrane of undifferentiated SSCs (PLZF was used as the marker) of mouse testes (A and B). PLD6 was mainly expressed on the cell membrane of murine cultured SSCs.

The cellular localization of PLD6 was further explored *via* cell and tissue immunofluoresence. As in Figure8A and B, PLZF was detected as a marker of undifferentiated SSCs 18. And PLD6 was uniquely expressed as the membrane protein in undifferentiated murine SSCs on post-partum day 2.5 and 28. As in Figure8C, PLD6 was mainly expressed on the cell membrane of murine cultured SSCs.

## Discussion

In this study, we used Shinohara's method to establish a long-term and stable culture of mouse SSCs with membrane-localized red fluorescence. At the time of writing this manuscript, we were able to maintain 32 passages of SSCs with stem cell activity, and the cells were frozen and thawed at least once. Exhibition of immunofluoresence by specific markers of undifferentiated SSCs, such as PLZF, GARA1 and OCT4, indicated that most of our SSCs were in an undifferentiated state. Further, transplantation of SSCs into busulfan-treated receipt mice produced vital offspring with membrane-localized red fluorescence in the testes and genetic labelling that could be stably transferred to F2 and F3 progeny. Thus, this successful *in vitro* mouse SSC culture provides us with a useful platform to study mouse SSCs at the molecular level.

We observed the red fluorescence was transmitted to 27%, not 100% of the pups after SSCs transplantation and mating. This is interesting but reasonable. The chemotherapeutic drug busulfan was chosen at a dose to treat recipient mice 33,34. However, a small number of endogenous stem cells persist and can reinitiate spermatogenesis. And with time, recovery of spermatogenesis becomes better. Therefore, it is helpful to use germ cells from donor containing a genetic marker 35. So during mating, only 27% of pups carried red fluorescence means about 1/3 seminiferous tubules were settled with transplanted stem cells and the other 2/3 may be reversible from busulfan-treated.

To establish the proteome profile for SSCs, we first constructed large-scale proteomes for MEF feeder cells and mouse SSCs co-cultured with MEFs by 2D-LC followed by LTQ Orbitrap Velos identification. This strategy is very simple and did not require the complicated purification of SSCs from the co-cultured cells. However, to detect more specific proteins of mouse SSCs, the cell suspension was plated on empty plates for approximately 3 hrs to enrich SSCs. We then compared the proteome profiles and focused on those proteins only expressed in SSCs; thus, the proteome profile was finally constructed with 682 proteins. Among them, 17 proteins (BCL6B, DMRT1, RARG, DNMT3B, EPCAM, FOXO1, PIWIL2, POU3F, RET, SALL4, SOHLH2, SOX3, TEX14, UTF1, ZBTB16, SMC6 and NFX2) have been reported to be involved in SSC self-renewal and differentiation 13,18,36–50. Among the genes for the 682 proteins identified, 278 were reported before in the gene expression profile of mouse SSCs 29,30. Therefore, we consider our protein list to be detailed enough and suitable for further investigation on mouse SSCs.

Bioinformatics analysis showed that 283 nucleoproteins accounted for about 41% of the total identified proteins. The STRING database was used to predict the interaction networks in the nucleoproteins. Further, according to Pathway studio analysis, 26 proteins were predicted to participate in RNA splicing, transcription initiation, DNA methylation, gene silencing and so on (DAVID). PATZ1, also named MAZR or ZSG, has recently been discovered to widely express a transcriptional regulatory factor that binds to the RING finger protein RNF4 51. Fedele *et al*. has demonstrated PATZ1 plays a critical role in spermatogenesis (expressed in primary spermatogonia, probably stem cells) and testicular tumourigenesis 52. However, the mechanism of how PATZ1 regulates SSC self-renewal needs to be further explored. These results were exciting because the classical intrinsic molecules that regulate the self-renewal of SSCs aretranscription factors, such as *Bcl6b*, *Etv5*, *Plzf*, *Ngn3*, *Taf4b* and so on. Here, we identified more candidate transcription factors regulating SSCs self-renew. Till date, there have been limitations in the information about interaction networks regulating SSC fate. We hope that this study will provide the resources required for more clearly understanding SSC self-renewal.

Studies on ESCs, hematopoietic stem cells and neuronal stem cells have identified several genes that are essential for stem cell proliferation and maintenance of pluripotency. Moreover, it has been suggested that several molecular mechanisms may be conserved among different types of stem cells 53. For example, LIN28 is a pluripotency factor that is highly expressed in pluripotent mouse ESCs 54, and it is also a marker for undifferentiated spermatogonia in the mouse 55. To focus on more reliable marker proteins for further exploration, we compared the proteomes established in our study with two known published proteomes of mouse ESCs 31,32: in total, 247 proteins in ESCs overlapped the ones identified in our study, and 30 proteins were found across all three studies. We narrowed the list down to three proteins that are reportedly involved in ESC pluripotency, in to explore whether they are also expressed in mouse SSCs.

The first protein, SALL1, is a multi-zinc finger transcription factor which acts as a transcriptional repressor that regulates kidney organogenesis 56. Karantzali *et al*. reported that SALL1 positively regulates NANOG expression and is a member of the transcriptional network that regulates pluripotency in mouse ESCs 57. The second one, EZH2, is the catalytic subunit of PRC2 (Polycomb repressive complex 2) and is highly expressed in ESCs 58. Villasante *et al*. reported that *Ezh2* directly regulates the epigenetic status of the *Nanog* promoter, which affects the balance of NANOG expression in ES/iPS cells and, therefore, the equilibrium between self-renewal and differentiation 59. With regard to the third protein, Yang *et al*. reported that RCOR2 is predominantly expressed in ESCs and forms a complex with histone demethylase LSD1. Knockdown of *Rcor2* in ESCs inhibited ESC proliferation and severely impaired their pluripotency 60. On the basis of the functions of these three proteins, we think that they might play important roles in maintaining mouse SSC self-renewal. Immunofluoresence localization showed that SALL1, EZH2 and RCOR2 are all highly expressed in the nucleus of mouse SSCs. Thus, future studies on SSCs should focus on these three proteins to explore the mechanisms that how they regulate SSC self-renewal.

Isolation and identification of SSCs from mammalian testes are essential to examine the mechanisms that regulate their functions. However, all markers for SSCs described to date are also expressed by other stem cells, such as ESCs, neural stem cells and HSCs. No marker described to date is expressed exclusively by SSCs in the testis; as a result, it is not possible to obtain pure SSC cultures. Thus, the SSC phenotype must be further characterized to identify definitive markers. Screening for an exclusive surface marker of SSCs is very important. With this aim in mind, we identified 131 predicted membrane proteins *via* proteomic technology. Among them, EPCAM is a reported surface antigen on SSCs 22,39. Potential specific surface markers of SSCs would be uniquely or highly expressed in mouse testis and SSCs. Therefore, the expression of each protein was analysed at the tissue level using the BioGPS database. Ten proteins with unique or high expression in mouse testes were further validated *via* RT-PCR. The results showed that all ten proteins were highly expressed in mouse testis and SSCs. Among them, *Gpat2* and *Pld6* were uniquely expressed in mouse testis. Next, PLD6 were verified to show strong membrane expression patterns in undifferentiated murine SSCs both *in vivo* and *in vitro*. Therefore, we consider that PLD6 is a potential novel marker of mouse SSCs. In future studies, we will explore whether Pld6 and others can be purified as a surface marker of mouse SSCs.

In conclusion, a specific proteome of mouse SSCs was constructed to understand the molecular mechanisms regulating SSC self-renewal and to identify surface markers for the purification of SSCs. The well-defined culture conditions and the transplantation assay used in this study provide a powerful system to analyse these intrinsic and surface proteins in mouse SSCs. The findings suggest the role of certain proteins in SSC self-renewal and as markers for SSCs. These proteins should be further explored to validate these findings and discover the mechanisms of SSC self-renewal.

## Supporting Information

**Table S1** The information of 682 proteins only expressed in SSCs.
