# Supplementary material for: Establishment of a proteome profile and identification of molecular markers for mouse spermatogonial stem cells
Source: J Cell Mol Med. 2014 Oct 29;19(3):521–34. doi: 10.1111/jcmm.12407 (PMC4369810; doi:10.1111/jcmm.12407)
Supplement: Supplementary file 1 [file jcmm0019-0521-sd1.doc]

| Ensembl | Gene names | Peptides SSC | Expression |
| --- | --- | --- | --- |
| ENSMUSG00000021255 | Esrrb | 1 | SSConly |
| ENSMUSG00000023403 | Stk31 | 10 | SSConly |
| ENSMUSG00000024642 | Tle4 | 1 | SSConly |
| ENSMUSG00000028565 | Nfia | 4 | SSConly |
| ENSMUSG00000036452 | Arhgap26 | 1 | SSConly |
| ENSMUSG00000049313 | Sorl1 | 2 | SSConly |
| ENSMUSG00000027412 | Lpin3 | 1 | SSConly |
| ENSMUSG00000017146 | Brca1 | 1 | SSConly |
| ENSMUSG00000039834 | Zfp335 | 1 | SSConly |
| ENSMUSG00000042408 | Zmym6 | 1 | SSConly |
| ENSMUSG00000037600 | 1810019J16Rik | 1 | SSConly |
| ENSMUSG00000043929 | Klhl15 | 1 | SSConly |
| ENSMUSG00000025384 | Faap100 | 1 | SSConly |
| ENSMUSG00000033233 | Trim45 | 1 | SSConly |
| ENSMUSG00000009108 | Gnat2 | 1 | SSConly |
| ENSMUSG00000025272 | Tro | 1 | SSConly |
| ENSMUSG00000040687 | Madd | 1 | SSConly |
| ENSMUSG00000027215 | Cd82 | 1 | SSConly |
| ENSMUSG00000049488 | Tmem67 | 1 | SSConly |
| ENSMUSG00000009566 | Fpgs | 1 | SSConly |
| ENSMUSG00000027379 | Bub1 | 1 | SSConly |
| ENSMUSG00000038705 | Gmeb2 | 1 | SSConly |
| ENSMUSG00000039715 | Wdr34 | 1 | SSConly |
| ENSMUSG00000017801 | Mlx | 1 | SSConly |
| ENSMUSG00000063019 | Manbal | 1 | SSConly |
| ENSMUSG00000000317 | Bcl6b | 1 | SSConly |
| ENSMUSG00000019952 | Poc1b | 1 | SSConly |
| ENSMUSG00000049128 | Ivl | 1 | SSConly |
| ENSMUSG00000049969 | Plekhf2 | 1 | SSConly |
| ENSMUSG00000028890 | Mtf1 | 1 | SSConly |
| ENSMUSG00000028784 | Spocd1 | 1 | SSConly |
| ENSMUSG00000027011 | Ube2e3 | 1 | SSConly |
| ENSMUSG00000024266 | Adad2 | 1 | SSConly |
| ENSMUSG00000046572 | Znf518b | 1 | SSConly |
| ENSMUSG00000033931 | Rbm34 | 1 | SSConly |
| ENSMUSG00000038495 | Otud7b | 1 | SSConly |
| ENSMUSG00000070420 | Zfp498 | 1 | SSConly |
| ENSMUSG00000027544 | Nfatc2 | 1 | SSConly |
| ENSMUSG00000037885 | Stk35 | 1 | SSConly |
| ENSMUSG00000027550 | Lrrcc1 | 1 | SSConly |
| ENSMUSG00000006019 | Dhx34 | 1 | SSConly |
| ENSMUSG00000062797 | l7Rn6 | 1 | SSConly |
| ENSMUSG00000008540 | Mgst1 | 1 | SSConly |
| ENSMUSG00000022487 | Gtsf1 | 1 | SSConly |
| ENSMUSG00000024948 | Map4k2 | 1 | SSConly |
| ENSMUSG00000028064 | Sema4a | 1 | SSConly |
| ENSMUSG00000040570 | Rundc3b | 1 | SSConly |
| ENSMUSG00000054312 | Mrps21 | 1 | SSConly |
| ENSMUSG00000052512 | Nav2 | 1 | SSConly |
| ENSMUSG00000040111 | Gramd1b | 1 | SSConly |
| ENSMUSG00000011267 | Zfp296 | 1 | SSConly |
| ENSMUSG00000066894 | Vsig10 | 1 | SSConly |
| ENSMUSG00000010051 | Hyal1 | 1 | SSConly |
| ENSMUSG00000074264 | Amy1 | 1 | SSConly |
| ENSMUSG00000020083 | 2010107G23Rik | 1 | SSConly |
| ENSMUSG00000022681 | Ntan1 | 1 | SSConly |
| ENSMUSG00000022972 | 1110004E09Rik | 1 | SSConly |
| ENSMUSG00000023051 | Tarbp2 | 1 | SSConly |
| ENSMUSG00000001288 | Rarg | 1 | SSConly |
| ENSMUSG00000047407 | Tgif1 | 1 | SSConly |
| ENSMUSG00000055874 | Foxi3 | 1 | SSConly |
| ENSMUSG00000061911 | Myt1l | 1 | SSConly |
| ENSMUSG00000022151 | Ttc33 | 1 | SSConly |
| ENSMUSG00000029454 | Mapkapk5 | 1 | SSConly |
| ENSMUSG00000067367 | Lyar | 1 | SSConly |
| ENSMUSG00000028617 | Lrrc42 | 1 | SSConly |
| ENSMUSG00000008140 | Inm02;2310044H10Rik | 1 | SSConly |
| ENSMUSG00000004018 | Fancl | 1 | SSConly |
| ENSMUSG00000002395 | Use1 | 1 | SSConly |
| ENSMUSG00000075411 | Bin2 | 1 | SSConly |
| ENSMUSG00000022790 | Igsf11 | 1 | SSConly |
| ENSMUSG00000063894 | Zfp192 | 1 | SSConly |
| ENSMUSG00000035629 | Kiaa0226;1700021K19Rik | 1 | SSConly |
| ENSMUSG00000034930 | Rtkn | 1 | SSConly |
| ENSMUSG00000024194 | Cuta | 1 | SSConly |
| ENSMUSG00000022472 | Pppde2 | 1 | SSConly |
| ENSMUSG00000019303 | Psmc3ip | 1 | SSConly |
| ENSMUSG00000020228 | Helb | 1 | SSConly |
| ENSMUSG00000030177 | Ccdc77 | 1 | SSConly |
| ENSMUSG00000089862 | Gm16039 | 1 | SSConly |
| ENSMUSG00000028483 | Snapc3 | 1 | SSConly |
| ENSMUSG00000006014 | Prg4 | 1 | SSConly |
| ENSMUSG00000021638 | Ocln | 1 | SSConly |
|  | Topaz1 | 1 | SSConly |
| ENSMUSG00000043323 | Fbrsl1 | 1 | SSConly |
| ENSMUSG00000033417 | 2700078E11Rik | 1 | SSConly |
| ENSMUSG00000034601 | 2700049A03Rik | 1 | SSConly |
| ENSMUSG00000066432 | Vmn2r68-ps | 1 | SSConly |
| ENSMUSG00000074238 | Ap1ar | 1 | SSConly |
| ENSMUSG00000022911 | Arl13b | 1 | SSConly |
| ENSMUSG00000022464 | Slc38a4 | 1 | SSConly |
| ENSMUSG00000025602 | Zfp202 | 1 | SSConly |
| ENSMUSG00000038425 | Poli | 1 | SSConly |
| ENSMUSG00000018425 | Dhx40 | 1 | SSConly |
| ENSMUSG00000068122 | Agtr2 | 1 | SSConly |
| ENSMUSG00000033961 | Zfp446 | 1 | SSConly |
| ENSMUSG00000051786 | Tubgcp6 | 1 | SSConly |
| ENSMUSG00000039789 | Zfp597 | 1 | SSConly |
| ENSMUSG00000037503 | Fam168b | 1 | SSConly |
| ENSMUSG00000074405 | Znf865;Zfp865 | 1 | SSConly |
| ENSMUSG00000059323 | Tonsl | 1 | SSConly |
| ENSMUSG00000074397 | Foxr1 | 1 | SSConly |
| ENSMUSG00000046111 | Kiaa1731;5830418K08Rik | 1 | SSConly |
| ENSMUSG00000021621 | Zcchc9 | 1 | SSConly |
| ENSMUSG00000007646 | Rad51c | 1 | SSConly |
| ENSMUSG00000023959 | Clic5 | 1 | SSConly |
| ENSMUSG00000037017 | Zscan21 | 1 | SSConly |
| ENSMUSG00000057699 | Tas2r131 | 1 | SSConly |
| ENSMUSG00000029068 | Ccnl2 | 1 | SSConly |
| ENSMUSG00000026162 | Nhej1 | 1 | SSConly |
| ENSMUSG00000023990 | Tfeb | 1 | SSConly |
| ENSMUSG00000014767 | Tbp | 1 | SSConly |
| ENSMUSG00000048834 | Vstm2a | 1 | SSConly |
| ENSMUSG00000038677 | Scube3 | 1 | SSConly |
| ENSMUSG00000021097 | Clmn | 1 | SSConly |
| ENSMUSG00000079020 | Slc45a4 | 1 | SSConly |
| ENSMUSG00000032815 | Fanca | 1 | SSConly |
| ENSMUSG00000029676 | Pot1a;Pot1 | 1 | SSConly |
| ENSMUSG00000000028 | Cdc45 | 1 | SSConly |
| ENSMUSG00000000552 | Zfp385a | 1 | SSConly |
| ENSMUSG00000053046 | Brsk2 | 1 | SSConly |
| ENSMUSG00000032921 | Odf4 | 1 | SSConly |
| ENSMUSG00000029112 | Nkx1-1 | 1 | SSConly |
| ENSMUSG00000034673 | Pbx2 | 1 | SSConly |
| ENSMUSG00000032113 | Chek1 | 1 | SSConly |
| ENSMUSG00000039431 | Mtmr7 | 1 | SSConly |
| ENSMUSG00000021418 | Rpp40 | 1 | SSConly |
| ENSMUSG00000074832 | 2410141K09Rik | 1 | SSConly |
| ENSMUSG00000067438 | Hmx1 | 1 | SSConly |
| ENSMUSG00000034845 | Plvap | 1 | SSConly |
| ENSMUSG00000047669 | Msl3l2 | 1 | SSConly |
| ENSMUSG00000024789 | Jak2 | 1 | SSConly |
| ENSMUSG00000024926 | Kat5 | 1 | SSConly |
| ENSMUSG00000053774 | Ubxn7 | 1 | SSConly |
| ENSMUSG00000035394 | Ccdc11 | 1 | SSConly |
| ENSMUSG00000026279 | Thap4 | 1 | SSConly |
| ENSMUSG00000027997 | Casp6 | 1 | SSConly |
| ENSMUSG00000070002 | Ell | 1 | SSConly |
| ENSMUSG00000032316 | Clk3 | 1 | SSConly |
| ENSMUSG00000006930 | Hap1 | 1 | SSConly |
| ENSMUSG00000057469 | E2f6 | 1 | SSConly |
| ENSMUSG00000050069 | Grem2 | 1 | SSConly |
| ENSMUSG00000027478 | Dnmt3b | 1 | SSConly |
| ENSMUSG00000031880 | Rrad | 1 | SSConly |
| ENSMUSG00000054626 | Xlr | 1 | SSConly |
| ENSMUSG00000026473 | Glul | 1 | SSConly |
| ENSMUSG00000025395 | Prim1 | 1 | SSConly |
| ENSMUSG00000018537 | Pcgf2 | 1 | SSConly |
| ENSMUSG00000033585 | Ndn | 1 | SSConly |
| ENSMUSG00000000149 | Gna12 | 1 | SSConly |
| ENSMUSG00000068267 | Cenpb | 1 | SSConly |
| ENSMUSG00000006678 | Pola1 | 1 | SSConly |
| ENSMUSG00000043895 | S1pr2 | 1 | SSConly |
| ENSMUSG00000020407 | Upp1 | 1 | SSConly |
| ENSMUSG00000028111 | Ctsk | 1 | SSConly |
| ENSMUSG00000021290 | Mp68 | 1 | SSConly |
| ENSMUSG00000045078 | Rnf216 | 1 | SSConly |
| ENSMUSG00000034450 | Gulo | 1 | SSConly |
| ENSMUSG00000038894 | Irs2 | 1 | SSConly |
| ENSMUSG00000086784 | Isoc2a | 1 | SSConly |
| ENSMUSG00000004642 | Slbp | 1 | SSConly |
| ENSMUSG00000027496 | Aurka | 1 | SSConly |
| ENSMUSG00000022865 | Cxadr | 1 | SSConly |
| ENSMUSG00000004655 | Aqp1 | 1 | SSConly |
| ENSMUSG00000075217 | Fads2p1 | 1 | SSConly |
| ENSMUSG00000027569 | Mrgbp;1600027N09Rik | 1 | SSConly |
| ENSMUSG00000048922 | Cdca2 | 1 | SSConly |
| ENSMUSG00000054003 | Tdrd9 | 1 | SSConly |
| ENSMUSG00000028952 | Zbtb48 | 1 | SSConly |
| ENSMUSG00000034773 |  | 1 | SSConly |
| ENSMUSG00000031811 | Fbxo31 | 1 | SSConly |
| ENSMUSG00000022763 | Aifm3 | 1 | SSConly |
| ENSMUSG00000031070 | Mrgprf | 1 | SSConly |
| ENSMUSG00000027936 | Crtc2 | 1 | SSConly |
| ENSMUSG00000039623 | Ap5z1;C330006K01Rik | 1 | SSConly |
| ENSMUSG00000074748 | Atxn7l3b | 1 | SSConly |
| ENSMUSG00000042726 | Trafd1 | 1 | SSConly |
| ENSMUSG00000062822 |  | 1 | SSConly |
| ENSMUSG00000045598 | Znf48 | 1 | SSConly |
| ENSMUSG00000036473 | Tbc1d24 | 1 | SSConly |
| ENSMUSG00000063804 | Lin28b | 1 | SSConly |
| ENSMUSG00000057788 | Ddx49 | 1 | SSConly |
| ENSMUSG00000021510 | A530054K11Rik | 1 | SSConly |
| ENSMUSG00000029433 | Diablo | 1 | SSConly |
| ENSMUSG00000044807 | Znf354c | 1 | SSConly |
| ENSMUSG00000069899;ENSMUSG00000073468 | Sft2d1 | 1 | SSConly |
| ENSMUSG00000043648 | Pld6 | 1 | SSConly |
| ENSMUSG00000045100 | Slc25a26 | 1 | SSConly |
| ENSMUSG00000000093 | Tbx2 | 1 | SSConly |
| ENSMUSG00000003665 | Has1 | 1 | SSConly |
| ENSMUSG00000030539 | Sema4b | 1 | SSConly |
| ENSMUSG00000009394 | Syn2 | 1 | SSConly |
| ENSMUSG00000055301 | Adh7 | 1 | SSConly |
| ENSMUSG00000024293 | Esco1 | 1 | SSConly |
| ENSMUSG00000046329 | Slc25a23 | 1 | SSConly |
| ENSMUSG00000031384 | Asb9 | 1 | SSConly |
| ENSMUSG00000030002 | Dusp11 | 1 | SSConly |
| ENSMUSG00000038214 | Bend3 | 1 | SSConly |
| ENSMUSG00000018548 | Trim37 | 1 | SSConly |
| ENSMUSG00000052915 | Msl1 | 1 | SSConly |
| ENSMUSG00000015759 | Cnih | 1 | SSConly |
| ENSMUSG00000025082 | Vwa2 | 1 | SSConly |
| ENSMUSG00000037363 | Letm2 | 1 | SSConly |
| ENSMUSG00000035275 | Raver2 | 1 | SSConly |
| ENSMUSG00000043257 | Pigv | 1 | SSConly |
| ENSMUSG00000020747 | Kiaa0195 | 1 | SSConly |
| ENSMUSG00000027751 | Fam48a | 1 | SSConly |
| ENSMUSG00000058093 | AA987161 | 1 | SSConly |
| ENSMUSG00000047635 |  | 1 | SSConly |
| ENSMUSG00000024519 | Cplx4 | 1 | SSConly |
| ENSMUSG00000034333 | Zbed4 | 1 | SSConly |
| ENSMUSG00000060950 | Trmt61a | 1 | SSConly |
| ENSMUSG00000058388 | Phtf1 | 1 | SSConly |
| ENSMUSG00000018347 | Znf18 | 1 | SSConly |
| ENSMUSG00000053730 | Tmem39b | 1 | SSConly |
| ENSMUSG00000022177 | Haus4 | 1 | SSConly |
| ENSMUSG00000033411 | Ctdspl2 | 1 | SSConly |
| ENSMUSG00000050428 | Fbxo46 | 1 | SSConly |
| ENSMUSG00000044134 | Fam109a | 1 | SSConly |
| ENSMUSG00000047518 | Slfnl1 | 1 | SSConly |
| ENSMUSG00000031371 | Haus7 | 1 | SSConly |
| ENSMUSG00000027339 | Rassf2 | 1 | SSConly |
| ENSMUSG00000034175 | Rhbdd3 | 1 | SSConly |
| ENSMUSG00000074682 | Zcchc3 | 1 | SSConly |
| ENSMUSG00000056832 | Ttc26 | 1 | SSConly |
| ENSMUSG00000041712 | Ubr7 | 1 | SSConly |
| ENSMUSG00000044847 | Lsm11 | 1 | SSConly |
| ENSMUSG00000043263 | Pyhin1 | 1 | SSConly |
| ENSMUSG00000040629 | Mael | 1 | SSConly |
| ENSMUSG00000034300 | Fam53c | 1 | SSConly |
| ENSMUSG00000056608 | Chd9 | 1 | SSConly |
| ENSMUSG00000029449 | Rhof | 1 | SSConly |
| ENSMUSG00000044066 | Cep68 | 1 | SSConly |
| ENSMUSG00000033364 | Usp37 | 1 | SSConly |
| ENSMUSG00000048285 | Frmd6 | 1 | SSConly |
| ENSMUSG00000031629 | Mlf1ip | 1 | SSConly |
| ENSMUSG00000045071 |  | 1 | SSConly |
| ENSMUSG00000037280 | Galnt6 | 1 | SSConly |
| ENSMUSG00000039985 | Fam60a | 1 | SSConly |
| ENSMUSG00000057229 | Atp5sl | 1 | SSConly |
| ENSMUSG00000020175 | Rab36 | 1 | SSConly |
| ENSMUSG00000048647 | Exd1 | 1 | SSConly |
| ENSMUSG00000025737 | Wdr24 | 1 | SSConly |
| ENSMUSG00000029246 | Ppat | 1 | SSConly |
| ENSMUSG00000051586;ENSMUSG00000003178 | Mical3 | 1 | SSConly |
| ENSMUSG00000022022 | Mtrf1 | 1 | SSConly |
| ENSMUSG00000031785 | Gpr56 | 1 | SSConly |
| ENSMUSG00000046573 | Lyrm4 | 1 | SSConly |
| ENSMUSG00000043155 | Hpdl | 1 | SSConly |
| ENSMUSG00000022833 | Ccdc14 | 1 | SSConly |
| ENSMUSG00000021953;ENSMUSG00000093611 | Tdh | 1 | SSConly |
| ENSMUSG00000028989 | Angptl7 | 1 | SSConly |
| ENSMUSG00000021519 | Mterfd1 | 1 | SSConly |
| ENSMUSG00000006585 | Cdt1 | 1 | SSConly |
| ENSMUSG00000019831 | Wasf1 | 1 | SSConly |
| ENSMUSG00000038555 | Reep2 | 1 | SSConly |
|  | Rfxap | 1 | SSConly |
| ENSMUSG00000029387 | Gtf2h3 | 1 | SSConly |
| ENSMUSG00000059586 | Nsmce2 | 1 | SSConly |
| ENSMUSG00000062309 | Rpp25 | 1 | SSConly |
| ENSMUSG00000071661 | Zbtb3 | 1 | SSConly |
| ENSMUSG00000007415 | Gatad1 | 1 | SSConly |
| ENSMUSG00000036114 | Rpp25l | 1 | SSConly |
| ENSMUSG00000036186 | Fam69b | 1 | SSConly |
| ENSMUSG00000024678 | Ms4a4d | 1 | SSConly |
| ENSMUSG00000032281 | Acsbg1 | 1 | SSConly |
| ENSMUSG00000070520 | Ndnl2 | 1 | SSConly |
| ENSMUSG00000062981 | Mrpl42 | 1 | SSConly |
| ENSMUSG00000028549 | Itgb3bp | 1 | SSConly |
| ENSMUSG00000035171 |  | 1 | SSConly |
| ENSMUSG00000029440 | Psmd9 | 1 | SSConly |
| ENSMUSG00000019689 |  | 1 | SSConly |
| ENSMUSG00000046942 | Mageb16 | 1 | SSConly |
| ENSMUSG00000039737 | Prkrip1 | 1 | SSConly |
| ENSMUSG00000024350 | Dnajc18 | 1 | SSConly |
| ENSMUSG00000024232 | Bambi | 1 | SSConly |
| ENSMUSG00000001707 | Eef1e1 | 1 | SSConly |
| ENSMUSG00000027794 | Sohlh2 | 1 | SSConly |
| ENSMUSG00000029270 | Fam69a | 1 | SSConly |
| ENSMUSG00000002797 | Ggct | 1 | SSConly |
| ENSMUSG00000031399 | Fam3a | 1 | SSConly |
| ENSMUSG00000020515 | Cnot8 | 1 | SSConly |
| ENSMUSG00000031898 | Dpep3 | 1 | SSConly |
| ENSMUSG00000005470 | Asf1b | 1 | SSConly |
| ENSMUSG00000036402 | Gng12 | 1 | SSConly |
| ENSMUSG00000038859 | Baiap2l1 | 1 | SSConly |
| ENSMUSG00000026594 | Ralgps2 | 1 | SSConly |
| ENSMUSG00000022033 | Pbk | 1 | SSConly |
| ENSMUSG00000030236 | Slco1b2 | 1 | SSConly |
| ENSMUSG00000052040 | Klf13 | 1 | SSConly |
| ENSMUSG00000028901 | Gmeb1 | 1 | SSConly |
| ENSMUSG00000038324 | Trpc4ap | 1 | SSConly |
| ENSMUSG00000035504 | Reep6 | 1 | SSConly |
| ENSMUSG00000062773 | Tex101 | 1 | SSConly |
| ENSMUSG00000038264 | Sema7a | 1 | SSConly |
| ENSMUSG00000048416 | Mlf1 | 1 | SSConly |
| ENSMUSG00000045969 | Ing1 | 1 | SSConly |
| ENSMUSG00000027048 | Abcb11 | 1 | SSConly |
| ENSMUSG00000045273 | Cenph | 1 | SSConly |
| ENSMUSG00000019877 | Serinc1 | 1 | SSConly |
| ENSMUSG00000005804 | Pldn | 1 | SSConly |
| ENSMUSG00000000276 | Dgke | 1 | SSConly |
| ENSMUSG00000010057 | Nprl2 | 1 | SSConly |
| ENSMUSG00000052544 | St6galnac3 | 1 | SSConly |
| ENSMUSG00000054667 | Irs4 | 1 | SSConly |
| ENSMUSG00000054115 | Skp2 | 1 | SSConly |
| ENSMUSG00000005148 | Klf5 | 1 | SSConly |
| ENSMUSG00000002289 | Angptl4 | 1 | SSConly |
| ENSMUSG00000039231 | Suv39h1 | 2 | SSConly |
| ENSMUSG00000079584 | Gm364 | 2 | SSConly |
| ENSMUSG00000058318 | Phf21a | 2 | SSConly |
| ENSMUSG00000036281 | Snapc4 | 2 | SSConly |
| ENSMUSG00000043411 | Usp48 | 2 | SSConly |
| ENSMUSG00000038598 | AI481877 | 2 | SSConly |
| ENSMUSG00000072980 | Oip5 | 2 | SSConly |
| ENSMUSG00000036959 | Bcorl1 | 2 | SSConly |
| ENSMUSG00000008855 | Hdac5 | 2 | SSConly |
| ENSMUSG00000079536 | Gm6880 | 2 | SSConly |
| ENSMUSG00000021087 | Rtn1 | 2 | SSConly |
| ENSMUSG00000038611 | Phrf1 | 2 | SSConly |
| ENSMUSG00000002058 | Unc119 | 2 | SSConly |
| ENSMUSG00000028799 | Zfp362 | 2 | SSConly |
| ENSMUSG00000073294 | AU022751 | 2 | SSConly |
| ENSMUSG00000017550 | Atad5 | 2 | SSConly |
| ENSMUSG00000073460 | Pnldc1 | 2 | SSConly |
| ENSMUSG00000062170 | Fmr1nb | 2 | SSConly |
| ENSMUSG00000090626 | Tex9 | 2 | SSConly |
| ENSMUSG00000079109 | Pms2 | 2 | SSConly |
| ENSMUSG00000073130 | Gm1141 | 2 | SSConly |
| ENSMUSG00000028944 | Prkag2 | 2 | SSConly |
| ENSMUSG00000068876 | Cgn | 2 | SSConly |
| ENSMUSG00000030411 | Nova2 | 2 | SSConly |
| ENSMUSG00000039968 | Rsbn1l | 2 | SSConly |
| ENSMUSG00000037221 | Mospd3 | 2 | SSConly |
| ENSMUSG00000068457 | Uty | 2 | SSConly |
| ENSMUSG00000027323 | Rad51 | 2 | SSConly |
| ENSMUSG00000029863 | Casp2 | 2 | SSConly |
| ENSMUSG00000020415 | Pttg1 | 2 | SSConly |
| ENSMUSG00000041957 | Pkp2 | 2 | SSConly |
| ENSMUSG00000040331 | Nsmce4a | 2 | SSConly |
| ENSMUSG00000024301 | Kifc5b | 2 | SSConly |
| ENSMUSG00000019866 | Aim1 | 2 | SSConly |
| ENSMUSG00000071369 | Map3k5 | 2 | SSConly |
| ENSMUSG00000008690 | Ncaph2;Gm7535 | 2 | SSConly |
| ENSMUSG00000026135 | Zfp142 | 2 | SSConly |
| ENSMUSG00000038116 | Phf20 | 2 | SSConly |
| ENSMUSG00000030110 | Ret | 2 | SSConly |
| ENSMUSG00000037325 | Bbs7 | 2 | SSConly |
| ENSMUSG00000033671 | Cep350 | 2 | SSConly |
| ENSMUSG00000023980 | Taf8 | 2 | SSConly |
| ENSMUSG00000021451 | Sema4d | 2 | SSConly |
| ENSMUSG00000049532 | Sall2 | 2 | SSConly |
| ENSMUSG00000044783 | Hjurp | 2 | SSConly |
| ENSMUSG00000058550 | Dppa4;Gm5501 | 2 | SSConly |
| ENSMUSG00000029381 | Shroom3 | 2 | SSConly |
| ENSMUSG00000026641 | Usf1;Usf2 | 2 | SSConly |
| ENSMUSG00000035293 | G2e3 | 2 | SSConly |
| ENSMUSG00000026098 | Pms1 | 2 | SSConly |
| ENSMUSG00000037795 | N4bp2 | 2 | SSConly |
| ENSMUSG00000021548 | Ccnh | 2 | SSConly |
| ENSMUSG00000041429 | Nthl1 | 2 | SSConly |
| ENSMUSG00000019986 | Ahi1 | 2 | SSConly |
| ENSMUSG00000037572 | Wdhd1 | 2 | SSConly |
| ENSMUSG00000048232 | Fbxo10 | 2 | SSConly |
| ENSMUSG00000026646 | Suv39h2 | 2 | SSConly |
| ENSMUSG00000020893 | Per1 | 2 | SSConly |
| ENSMUSG00000071226 | Cecr2 | 2 | SSConly |
| ENSMUSG00000030491 | Tdrd12 | 2 | SSConly |
| ENSMUSG00000059554 | Ccdc28a | 2 | SSConly |
| ENSMUSG00000022394 | L3mbtl2;Mbtd1 | 2 | SSConly |
| ENSMUSG00000003680 | Taf6l | 2 | SSConly |
| ENSMUSG00000024174 | Pot1b | 2 | SSConly |
| ENSMUSG00000030271 | Ogg1 | 2 | SSConly |
| ENSMUSG00000030722 | Nfatc2ip | 2 | SSConly |
| ENSMUSG00000027359 | Slc27a2 | 2 | SSConly |
| ENSMUSG00000020471 | Pold2 | 2 | SSConly |
| ENSMUSG00000051510 | Mafg | 2 | SSConly |
| ENSMUSG00000026239 | Pde6d | 2 | SSConly |
| ENSMUSG00000040009 | Gnaz | 2 | SSConly |
| ENSMUSG00000058454 | Dhcr7 | 2 | SSConly |
| ENSMUSG00000056436 | Cyct | 2 | SSConly |
| ENSMUSG00000074207 | Adh1 | 2 | SSConly |
| ENSMUSG00000038692 | Hoxb4 | 2 | SSConly |
| ENSMUSG00000032035 | Ets1 | 2 | SSConly |
| ENSMUSG00000025577 | Cbx2 | 2 | SSConly |
| ENSMUSG00000022548 | Apod | 2 | SSConly |
| ENSMUSG00000037361 | Sf3b14 | 2 | SSConly |
| ENSMUSG00000028702 | Rad54l | 2 | SSConly |
| ENSMUSG00000001942 | Siae | 2 | SSConly |
| ENSMUSG00000038766 | Gabpb2 | 2 | SSConly |
| ENSMUSG00000033882 | Rbm46 | 2 | SSConly |
| ENSMUSG00000059669 | Taf1b | 2 | SSConly |
| ENSMUSG00000030867 | Plk1 | 2 | SSConly |
| ENSMUSG00000040856 | Dlk1 | 2 | SSConly |
| ENSMUSG00000026669 | Mcm10 | 2 | SSConly |
| ENSMUSG00000049232 | Tigd2 | 2 | SSConly |
| ENSMUSG00000059920 | 4930453N24Rik | 2 | SSConly |
| ENSMUSG00000001786 | Fbxo7 | 2 | SSConly |
| ENSMUSG00000001569 | Nom1 | 2 | SSConly |
| ENSMUSG00000040007 | Bahd1 | 2 | SSConly |
| ENSMUSG00000031333 | Abcb7 | 2 | SSConly |
| ENSMUSG00000055782 | Abcd2 | 2 | SSConly |
| ENSMUSG00000023018 | Smarcd1 | 2 | SSConly |
| ENSMUSG00000016494 | Cd34 | 2 | SSConly |
| ENSMUSG00000061589 | Dot1l | 2 | SSConly |
| ENSMUSG00000039621 | Prex1 | 2 | SSConly |
| ENSMUSG00000054770 | Kctd18 | 2 | SSConly |
| ENSMUSG00000001517 | Foxm1 | 2 | SSConly |
| ENSMUSG00000031665 | Sall1 | 2 | SSConly |
| ENSMUSG00000012443 | Kif11 | 2 | SSConly |
| ENSMUSG00000059851 | Suv420h2 | 2 | SSConly |
| ENSMUSG00000060176 | Kif27 | 2 | SSConly |
| ENSMUSG00000024817 | Uhrf2 | 2 | SSConly |
| ENSMUSG00000005225 | Plekha8 | 2 | SSConly |
| ENSMUSG00000035919 | Bbs9 | 2 | SSConly |
| ENSMUSG00000041444 | Arhgap32 | 2 | SSConly |
| ENSMUSG00000005682 | Pan2 | 2 | SSConly |
| ENSMUSG00000026107 | Obfc2a | 2 | SSConly |
| ENSMUSG00000030726 | Pold3 | 2 | SSConly |
| ENSMUSG00000047989 | Ino80c | 2 | SSConly |
| ENSMUSG00000024604 | Rbm22 | 2 | SSConly |
| ENSMUSG00000039089 | L3mbtl3 | 2 | SSConly |
| ENSMUSG00000024220 | Znf76 | 2 | SSConly |
| ENSMUSG00000025235 | Bbs4 | 2 | SSConly |
| ENSMUSG00000047115 | Fam221a | 2 | SSConly |
| ENSMUSG00000053070 | 9230110C19Rik | 2 | SSConly |
| ENSMUSG00000042148 | Cox10 | 2 | SSConly |
| ENSMUSG00000037316 | Bag4 | 2 | SSConly |
| ENSMUSG00000063268 | Parp10 | 2 | SSConly |
| ENSMUSG00000021411 | Pxdc1;1300014I06Rik | 2 | SSConly |
| ENSMUSG00000021113 | Snapc1 | 2 | SSConly |
| ENSMUSG00000031262 | Cenpi | 2 | SSConly |
| ENSMUSG00000022179 |  | 2 | SSConly |
| ENSMUSG00000039810 | Zc3h10 | 2 | SSConly |
| ENSMUSG00000018846 | Pank3;Pank2 | 2 | SSConly |
| ENSMUSG00000018733 | Pex12 | 2 | SSConly |
| ENSMUSG00000041346 | Wrap53 | 2 | SSConly |
| ENSMUSG00000056973 | Ces1d | 2 | SSConly |
| ENSMUSG00000025608 | Podxl | 2 | SSConly |
| ENSMUSG00000042167 | Papd4 | 2 | SSConly |
| ENSMUSG00000024791 | Cdca5 | 2 | SSConly |
| ENSMUSG00000022774 | Ncbp2 | 2 | SSConly |
| ENSMUSG00000020171 | Yeats4 | 2 | SSConly |
| ENSMUSG00000021391 | Cenpp | 2 | SSConly |
| ENSMUSG00000021958 | Pinx1 | 2 | SSConly |
| ENSMUSG00000055612 | Cdca7 | 2 | SSConly |
| ENSMUSG00000022945 | Chaf1b | 2 | SSConly |
| ENSMUSG00000003208 | Ccdc94 | 2 | SSConly |
| ENSMUSG00000026955 |  | 2 | SSConly |
| ENSMUSG00000079553 | Kifc1 | 2 | SSConly |
| ENSMUSG00000002012 | Pnck | 2 | SSConly |
| ENSMUSG00000030538 | Cib1 | 2 | SSConly |
| ENSMUSG00000050107 | Gsg2 | 2 | SSConly |
| ENSMUSG00000024193 | Phf1 | 2 | SSConly |
| ENSMUSG00000031431 | Tsc22d3 | 2 | SSConly |
| ENSMUSG00000034621 | Gpatch8 | 3 | SSConly |
| ENSMUSG00000045179 | Sox3 | 3 | SSConly |
| ENSMUSG00000027469 | Tpx2 | 3 | SSConly |
| ENSMUSG00000027242 | Wdr76 | 3 | SSConly |
| ENSMUSG00000056486 | Chn1 | 3 | SSConly |
| ENSMUSG00000024201 | Kdm4b | 3 | SSConly |
| ENSMUSG00000041268 | Dmxl2 | 3 | SSConly |
| ENSMUSG00000038453 | Srcin1 | 3 | SSConly |
| ENSMUSG00000031253 | Srpx2 | 3 | SSConly |
| ENSMUSG00000008489 | Elavl2;Elavl4 | 3 | SSConly |
| ENSMUSG00000031644 | Nek1 | 3 | SSConly |
| ENSMUSG00000031921 | Terf2 | 3 | SSConly |
| ENSMUSG00000032307 | Ube2q2 | 3 | SSConly |
| ENSMUSG00000063808 | Gpatch1 | 3 | SSConly |
| ENSMUSG00000090083 | Rnf8 | 3 | SSConly |
| ENSMUSG00000040669 | Phc1 | 3 | SSConly |
| ENSMUSG00000070822 | Zscan18 | 3 | SSConly |
| ENSMUSG00000038664 | Herc1 | 3 | SSConly |
| ENSMUSG00000090946 | 2010109K11Rik | 3 | SSConly |
| ENSMUSG00000038393 | Txnip | 3 | SSConly |
| ENSMUSG00000022016 | Akap11 | 3 | SSConly |
| ENSMUSG00000000148 | Baat1;Brat1 | 3 | SSConly |
| ENSMUSG00000014850 | Msh3 | 3 | SSConly |
| ENSMUSG00000032405 | Pias1 | 3 | SSConly |
| ENSMUSG00000039716 | Dock3 | 3 | SSConly |
| ENSMUSG00000040728 | Esrp1 | 3 | SSConly |
| ENSMUSG00000024795 | Kif20b | 3 | SSConly |
| ENSMUSG00000032413 | Rasa2 | 3 | SSConly |
| ENSMUSG00000026788 | Zbtb43 | 3 | SSConly |
| ENSMUSG00000021175 | Cdca7l | 3 | SSConly |
| ENSMUSG00000024747 | Aldh1a7 | 3 | SSConly |
| ENSMUSG00000017009 | Sdc4 | 3 | SSConly |
| ENSMUSG00000001524 | Gtf2h4 | 3 | SSConly |
|  | H2-K1 | 3 | SSConly |
| ENSMUSG00000034311 | Kif4 | 3 | SSConly |
| ENSMUSG00000016194 | Hsd11b1 | 3 | SSConly |
| ENSMUSG00000030094 | Xpc | 3 | SSConly |
|  | Rasa2 | 3 | SSConly |
| ENSMUSG00000025586 | Cpeb1 | 3 | SSConly |
| ENSMUSG00000029591 | Ung | 3 | SSConly |
| ENSMUSG00000069089 | Cdk7 | 3 | SSConly |
| ENSMUSG00000036211 | Hist1h1t | 3 | SSConly |
| ENSMUSG00000038010 | Ccdc138 | 3 | SSConly |
| ENSMUSG00000001138 | Cnnm3 | 3 | SSConly |
| ENSMUSG00000073177 | Gm773 | 3 | SSConly |
| ENSMUSG00000029134 | Plb1 | 3 | SSConly |
| ENSMUSG00000032936 | Camkv | 3 | SSConly |
| ENSMUSG00000022623 | Shank3 | 3 | SSConly |
| ENSMUSG00000040620 | Dhx33 | 3 | SSConly |
| ENSMUSG00000043384 | Gprasp1 | 3 | SSConly |
| ENSMUSG00000018143 | Mafk | 3 | SSConly |
| ENSMUSG00000035683 | Melk | 3 | SSConly |
| ENSMUSG00000026803 | Ttf1 | 3 | SSConly |
| ENSMUSG00000001948 | Spa17 | 3 | SSConly |
| ENSMUSG00000029283 | Cdc7 | 3 | SSConly |
| ENSMUSG00000030811 | Fbxl19 | 3 | SSConly |
| ENSMUSG00000025266 | Gnl3l | 3 | SSConly |
| ENSMUSG00000021182 | Ccdc88c | 3 | SSConly |
| ENSMUSG00000048799 | Cep120 | 3 | SSConly |
| ENSMUSG00000034032 | Rpap1 | 3 | SSConly |
| ENSMUSG00000003847 | Nfat5 | 3 | SSConly |
| ENSMUSG00000004667 | Polr2e | 3 | SSConly |
| ENSMUSG00000044927 | H1fx | 3 | SSConly |
| ENSMUSG00000027018 | Hat1 | 3 | SSConly |
| ENSMUSG00000024240 | Epc1 | 3 | SSConly |
| ENSMUSG00000026274 | Pask | 3 | SSConly |
| ENSMUSG00000045822 | Zswim3 | 3 | SSConly |
| ENSMUSG00000020227 | Irak3 | 3 | SSConly |
| ENSMUSG00000069678 | Pcgf1 | 3 | SSConly |
| ENSMUSG00000004947 | Dtx2 | 3 | SSConly |
| ENSMUSG00000040446 | Rprd1a | 3 | SSConly |
| ENSMUSG00000039178 | Tbc1d19 | 3 | SSConly |
| ENSMUSG00000045394 | Epcam | 3 | SSConly |
| ENSMUSG00000041805 | Pramel1 | 3 | SSConly |
| ENSMUSG00000046180 | 4930550L24Rik | 3 | SSConly |
| ENSMUSG00000023919 | Cenpq | 3 | SSConly |
| ENSMUSG00000055067 | Smyd3 | 3 | SSConly |
| ENSMUSG00000028089 | Chd1l | 3 | SSConly |
| ENSMUSG00000022748 |  | 3 | SSConly |
| ENSMUSG00000009596 | Taf7l | 3 | SSConly |
| ENSMUSG00000025480 | Syce1 | 3 | SSConly |
| ENSMUSG00000054727 | 1700013H16Rik | 3 | SSConly |
| ENSMUSG00000027340 | Slc23a2 | 3 | SSConly |
| ENSMUSG00000036442 | Thap11 | 3 | SSConly |
| ENSMUSG00000033765 | Calm4 | 3 | SSConly |
| ENSMUSG00000030041 | D6Mm5e | 3 | SSConly |
| ENSMUSG00000043872 | Zmym1 | 4 | SSConly |
| ENSMUSG00000029050 | Ski | 4 | SSConly |
| ENSMUSG00000038773 | Kdm3b | 4 | SSConly |
| ENSMUSG00000022364 | Wdr67 | 4 | SSConly |
| ENSMUSG00000029687 | Ezh2 | 4 | SSConly |
| ENSMUSG00000024169 | Ift140 | 4 | SSConly |
| ENSMUSG00000032376 | Usp3 | 4 | SSConly |
| ENSMUSG00000039990 | 2700050L05Rik;Edrf1 | 4 | SSConly |
| ENSMUSG00000078236 | Pou3f1 | 4 | SSConly |
| ENSMUSG00000025105 | Bnc1 | 4 | SSConly |
| ENSMUSG00000031540 | Myst3;Kat6a | 4 | SSConly |
| ENSMUSG00000036023 | Parp2 | 4 | SSConly |
| ENSMUSG00000030528 | Blm | 4 | SSConly |
| ENSMUSG00000028766 | Alpl | 4 | SSConly |
| ENSMUSG00000028271 | Gtf2b | 4 | SSConly |
| ENSMUSG00000020092 | Pald | 4 | SSConly |
| ENSMUSG00000006920 | Ezh1 | 4 | SSConly |
| ENSMUSG00000009670 | Tex11 | 4 | SSConly |
| ENSMUSG00000052056 | Zfp217 | 4 | SSConly |
| ENSMUSG00000064128 | Cenpj | 4 | SSConly |
| ENSMUSG00000033799 | Fam208b | 4 | SSConly |
| ENSMUSG00000019564 | Arid3a | 4 | SSConly |
| ENSMUSG00000010592 | Dazl | 4 | SSConly |
| ENSMUSG00000031832 | Taf1c | 4 | SSConly |
| ENSMUSG00000025764 | Phf17 | 4 | SSConly |
| ENSMUSG00000020387 | Phf15 | 4 | SSConly |
| ENSMUSG00000042195 | Slc35f2 | 4 | SSConly |
| ENSMUSG00000022949 | Clic6 | 4 | SSConly |
| ENSMUSG00000047539 | Fbxo28 | 4 | SSConly |
| ENSMUSG00000036912 | Piwil4 | 4 | SSConly |
| ENSMUSG00000000085 | Scmh1 | 4 | SSConly |
| ENSMUSG00000063810 | Alms1 | 4 | SSConly |
| ENSMUSG00000040013 | Fkbp6 | 4 | SSConly |
| ENSMUSG00000022434 | Fam118a | 4 | SSConly |
| ENSMUSG00000027353 | Mcm8 | 4 | SSConly |
| ENSMUSG00000032101 | Ddx25 | 4 | SSConly |
| ENSMUSG00000044167 | Foxo1 | 4 | SSConly |
| ENSMUSG00000040506 | Ambra1 | 5 | SSConly |
| ENSMUSG00000040524 | Znf609;Zfp609 | 5 | SSConly |
| ENSMUSG00000029267 | Mtf2 | 5 | SSConly |
| ENSMUSG00000032999 | Nlrp4f | 5 | SSConly |
| ENSMUSG00000038518 | Jarid2 | 5 | SSConly |
| ENSMUSG00000039457 | Ppl | 5 | SSConly |
| ENSMUSG00000003545 | Fosb | 5 | SSConly |
| ENSMUSG00000047674 | Pdha2 | 5 | SSConly |
| ENSMUSG00000040181 | Fmo1 | 5 | SSConly |
| ENSMUSG00000039849 | Pcif1 | 5 | SSConly |
| ENSMUSG00000016253 | Th1l | 5 | SSConly |
| ENSMUSG00000020453 | Patz1 | 5 | SSConly |
| ENSMUSG00000045545 | Krt14 | 5 | SSConly |
| ENSMUSG00000026383 | Epb41l5 | 5 | SSConly |
| ENSMUSG00000022529 | Zfp263 | 5 | SSConly |
| ENSMUSG00000024943 | Smc5 | 5 | SSConly |
| ENSMUSG00000019992 | Fam54a | 5 | SSConly |
| ENSMUSG00000000441 | Raf1 | 5 | SSConly |
| ENSMUSG00000029363 | Rfc5 | 5 | SSConly |
| ENSMUSG00000027315 | Spint1 | 5 | SSConly |
| ENSMUSG00000039994 | Timeless | 5 | SSConly |
| ENSMUSG00000004661 | Arid3b | 5 | SSConly |
| ENSMUSG00000031303 | Map3k15 | 6 | SSConly |
| ENSMUSG00000034023 | Fancd2 | 6 | SSConly |
| ENSMUSG00000028487 | Bnc2 | 6 | SSConly |
| ENSMUSG00000038379 | Ttk | 6 | SSConly |
| ENSMUSG00000026691 | Fmo3 | 6 | SSConly |
| ENSMUSG00000036672 | Cenpt | 6 | SSConly |
| ENSMUSG00000021569 | Trip13 | 6 | SSConly |
| ENSMUSG00000010277 | Kiaa0100 | 6 | SSConly |
| ENSMUSG00000041817 | Fam169a | 6 | SSConly |
| ENSMUSG00000047751 | Utf1 | 6 | SSConly |
| ENSMUSG00000073125 | Xlr3b | 6 | SSConly |
| ENSMUSG00000019214 | Chtf18 | 6 | SSConly |
| ENSMUSG00000022034 | Esco2 | 6 | SSConly |
| ENSMUSG00000039187 | Fanci | 6 | SSConly |
| ENSMUSG00000000365 | Rnf17 | 6 | SSConly |
| ENSMUSG00000019338 | Znf687 | 6 | SSConly |
| ENSMUSG00000040928 | S100pbp | 6 | SSConly |
| ENSMUSG00000041577 | Prelp | 6 | SSConly |
| ENSMUSG00000029012 | Orc5 | 6 | SSConly |
| ENSMUSG00000007080 | Pole | 6 | SSConly |
| ENSMUSG00000035427;ENSMUSG00000082033 | Mageb4;Mageb10-ps | 7 | SSConly |
| ENSMUSG00000040434 | Gyltl1b | 7 | SSConly |
| ENSMUSG00000044433 | Camsap3 | 7 | SSConly |
| ENSMUSG00000046774 | 8030474K03Rik | 7 | SSConly |
| ENSMUSG00000022387 | Brd1 | 7 | SSConly |
| ENSMUSG00000073434 | Wdr90 | 7 | SSConly |
| ENSMUSG00000046691 | Chtf8 | 7 | SSConly |
| ENSMUSG00000058290 | Espl1 | 7 | SSConly |
| ENSMUSG00000020156 | Mum1 | 7 | SSConly |
| ENSMUSG00000026806 | Ddx31 | 7 | SSConly |
| ENSMUSG00000044595 | Dnd1 | 7 | SSConly |
| ENSMUSG00000035024 | Ncapd3 | 7 | SSConly |
| ENSMUSG00000029703 | LRWD1 | 7 | SSConly |
| ENSMUSG00000044149 | Nkrf | 7 | SSConly |
| ENSMUSG00000020677 | Ddx52 | 7 | SSConly |
| ENSMUSG00000004100 | Ppan | 7 | SSConly |
| ENSMUSG00000022432 | Smc1b | 7 | SSConly |
| ENSMUSG00000020608 | Smc6 | 7 | SSConly |
| ENSMUSG00000073894 | Rbmxl2 | 7 | SSConly |
| ENSMUSG00000023104 | Rfc2 | 7 | SSConly |
| ENSMUSG00000023015 | Racgap1 | 7 | SSConly |
| ENSMUSG00000057156 | Homez | 8 | SSConly |
| ENSMUSG00000020681 | Ace | 8 | SSConly |
| ENSMUSG00000002190 | Clgn | 8 | SSConly |
| ENSMUSG00000034690 | Nlrp4c | 8 | SSConly |
| ENSMUSG00000047193 | Dync2h1 | 8 | SSConly |
| ENSMUSG00000050424 | Pnma5 | 8 | SSConly |
|  | Tdrd5 | 8 | SSConly |
| ENSMUSG00000051220 | Ercc6l | 8 | SSConly |
| ENSMUSG00000028873 | Cdca8 | 8 | SSConly |
| ENSMUSG00000024837 | Dmrt1 | 8 | SSConly |
| ENSMUSG00000034171 | Faah | 9 | SSConly |
| ENSMUSG00000066687 | Zbtb16 | 9 | SSConly |
| ENSMUSG00000053470 | Kdm3a | 9 | SSConly |
| ENSMUSG00000010342 | Tex14 | 9 | SSConly |
| ENSMUSG00000039354 | Smarcal1 | 9 | SSConly |
| ENSMUSG00000040170 | Fmo2 | 9 | SSConly |
| ENSMUSG00000036928 | Stag3 | 10 | SSConly |
| ENSMUSG00000009941 | Nxf2 | 10 | SSConly |
| ENSMUSG00000010796 | Asz1 | 10 | SSConly |
| ENSMUSG00000028977 | Casz1 | 11 | SSConly |
| ENSMUSG00000015365 | Mov10l1 | 11 | SSConly |
| ENSMUSG00000024135 | Srbd1 | 11 | SSConly |
| ENSMUSG00000024968 | Rcor2 | 11 | SSConly |
| ENSMUSG00000040044 | Orc3 | 11 | SSConly |
| ENSMUSG00000046338 | Gpat2 | 12 | SSConly |
| ENSMUSG00000079259 | Trim71 | 12 | SSConly |
| ENSMUSG00000061186 | Sfmbt2 | 12 | SSConly |
| ENSMUSG00000070732 | Rbm44 | 12 | SSConly |
| ENSMUSG00000027547 | Sall4 | 12 | SSConly |
| ENSMUSG00000043065 | Spice1 | 12 | SSConly |
| ENSMUSG00000028587 | Orc1 | 13 | SSConly |
| ENSMUSG00000038644 | Pold1 | 13 | SSConly |
| ENSMUSG00000021758 | Ddx4 | 13 | SSConly |
| ENSMUSG00000025081 | Tdrd1 | 14 | SSConly |
| ENSMUSG00000021311 | Mtr | 15 | SSConly |
| ENSMUSG00000029191 | Rfc1 | 15 | SSConly |
| ENSMUSG00000025001 | Hells | 16 | SSConly |
| ENSMUSG00000001228 | Uhrf1 | 16 | SSConly |
| ENSMUSG00000033644 | Piwil2 | 18 | SSConly |
| ENSMUSG00000036202 | Rif1 | 23 | SSConly |
| ENSMUSG00000030091 | Nup210 | 26 | SSConly |
